# Understanding the Abnormal Today for Life Tomorrow: A Study of the Generations During the COVID-19 Pandemic

**DOI:** 10.1093/geroni/igab046.308

**Published:** 2021-12-17

**Authors:** Lisa D'Ambrosio, Lisa D'Ambrosio

## Abstract

The spread of COVID-19 in the United States in early 2020 abruptly transformed American life, with widespread closures of public spaces and businesses, limitations on social activities, and the need for individuals to physically distance from each other. Some changes wrought by the virus may persist post-pandemic - such as Americans' adoption of new technologies or disease prevention behaviors. Since the onset of COVID-related safer-at-home orders, the MIT AgeLab has sought to understand how the pandemic affects people’s attitudes and behaviors. This symposium will present findings drawn from three waves of national, online surveys conducted in 2020: March (N=1202), May-June (N=1,387), and November-December 2020 (N=1444). The surveys explored participants’ COVID-19-related attitudes and behaviors across a range of domains. Each presentation in this symposium will highlight a different focus of cross-generational research conducted over time, with a particular focus on experiences of adults ages 55 and over. The first will focus on participants’ overall health, wellbeing, and perceptions of the COVID-19 vaccine. The second will present experiences of family caregivers of older adults and children. The third will center on the impact of the pandemic on the generations’ retirement and longevity planning experiences. The fourth and final presentation will focus on participants’ attitudes and experiences using and adopting technology. This symposium will deepen attendees’ understandings of multigenerational attitudes and experiences during the COVID-19 pandemic, with a particular focus on the experiences of adults ages 55 and over.

